# Inhibition of Aurora A enhances radiosensitivity in selected lung cancer cell lines

**DOI:** 10.1186/s12931-019-1194-8

**Published:** 2019-10-23

**Authors:** Ningbo Liu, Yong Antican Wang, Yunguang Sun, Jeffrey Ecsedy, Jifeng Sun, Xue Li, Ping Wang

**Affiliations:** 1 Department of Radiation Oncology, Tianjin Medical University Cancer Institute and Hospital, Oncology Key Laboratory of Cancer Prevention and Therapy, National Clinical Research Center of Cancer, Tianjin, 300060 China; 2 Biomed Innovation Center of Yehoo Group Co. Ltd., Shenzhen, 518000 China; 3 Department of Radiation Oncology, Thomas Jefferson University, Philadelphia, PA USA; 4 Department of Pathology, Medical College of Wisconsin, Milwaukee, WI USA; 5 Takeda Pharmaceuticals International Co, Cambridge, MA UK

**Keywords:** AURKA, MLN8237, Alisertib, Radiosensitivity, Lung cancer, P53

## Abstract

**Background:**

In mammalian cells, Aurora serine/threonine kinases (Aurora A, B, and C) are expressed in a cell cycle-dependent fashion as key mitotic regulators required for the maintenance of chromosomal stability. Aurora-A (AURKA) has been proven to be an oncogene in a variety of cancers; however, whether its expression relates to patient survival and the association with radiotherapy remains unclear in non-small cell lung cancer (NSCLC).

**Methods:**

Here, we first analyzed AURKA expression in 63 NSCLC tumor samples by immunohistochemistry (IHC) and used an MTS assay to compare cell survival by targeting AURKA with MLN8237 (Alisertib) in H460 and HCC2429 (P53-competent), and H1299 (P53-deficient) cell lines. The radiosensitivity of MLN8237 was further evaluated by clonogenic assay. Finally, we examined the effect of combining radiation and AURKA inhibition in vivo with a xenograft model and explored the potential mechanism.

**Results:**

We found that increased AURKA expression correlated with decreased time to progression and overall survival (*p* = 0.0447 and 0.0096, respectively). AURKA inhibition using 100 nM MLN8237 for 48 h decreases cell growth in a partially P53-dependent manner, and the survival rates of H460, HCC2429, and H1299 cells were 56, 50, and 77%, respectively. In addition, the survival of H1299 cells decreased 27% after ectopic restoration of P53 expression, and the radiotherapy enhancement was also influenced by P53 expression (DER H460 = 1.33; HCC2429 = 1.35; H1299 = 1.02). Furthermore, tumor growth of H460 was delayed significantly in a subcutaneous mouse model exposed to both MLN8237 and radiation.

**Conclusions:**

Taken together, our results confirmed that the expression of AURKA correlated with decreased NSCLC patient survival, and it might be a promising inhibition target when combined with radiotherapy, especially for P53-competent lung cancer cells. Modulation of P53 function could provide a new option for reversing cell resistance to the AURKA inhibitor MLN8237, which deserves further investigation.

## Background

Lung cancer is responsible for the most cancer-related deaths for both men and women throughout the world [1]. The American Cancer Society estimated that approximately 234,030 new cases of lung cancer would be diagnosed, and approximately 154,050 deaths due to lung cancer would occur in 2018 [2]. Although radiotherapy is the most important nonsurgical treatment for NSCLC, the efficacy of radiation is still limited by various factors [3], for example, the intrinsic radiation resistance of several tumors, the heterogenicity of tumor cells, the tumor microenvironment and the immune response [4–6]. It is well known that the cell division stage (M-phase) has the highest sensitivity to ionizing radiation in some cell types [7]. Therefore, new strategies to combine radiotherapy with agents that specifically target key factors of the cell cycle are currently being tested [8, 9].

The serine-threonine kinase family (Aurora kinase, A, B, and C) plays integral roles in centrosome maturation, chromosome segregation, and cytokinesis [10–12]. Overexpression of Aurora kinase is associated with tumorigenesis in multiple solid tumor types, including non-small cell lung cancer [13–16], and has been implicated in the development of resistance to chemotherapy [17, 18]. Aurora kinase A (AURKA), which has been implicated in several vital events in mitosis [19, 20], can physically associate with several important proteins, including P53 and BRCA1 [21, 22]. AURKA phosphorylates P53 at Ser-315 and reduces the transcriptional activity of P53 via regulation of P53 stability [23], while the phosphorylation of P53 at Ser-215 abrogates P53 DNA binding and transactivation activity. Downstream target genes of P53, such as p21^Cip/WAF1^ and PTEN, were inhibited by AURKA in this Ser-215 phosphorylation-dependent manner and were not affected by phosphorylation of Ser-315 [24]. On the other hand, P53 can negatively regulate AURKA via both transcriptional and posttranslational regulation. Knocking down P53 allows increased AURKA expression by removing the block on transcription factor E2F3 from binding the AURKA promoter. In addition, normal P53 activity includes downregulating Fbxw7, part of the ubiquitin ligase complex, which is responsible for targeting AURKA for destruction [25].

AURKA inhibition has been shown to increase chemosensitivity in lung and ovarian cancer cell lines [17, 26] and has been suggested as a mechanism to increase radiosensitivity in non-small cell lung cancer [27], as well as other cancers [28, 29]. Currently, Aurora kinase inhibitors have been extensively developed and characterized as compounds that block cell-cycle progression and induce apoptosis in a diverse range of human tumor types [30–32]. MLN8237 (also known as alisertib), a second-generation orally bioavailable inhibitor of AURKA, is being investigated for treating advanced malignancies due to both its in vitro and in vivo activities against a broad range of tumor types [33–35].

It has been well established that P53 functions as a tumor suppressor in various cancers and can modulate chemo- and radiotherapy sensitivity in vitro [36]. Mutations in P53 result in loss of its normal anti-proliferative functions in a dominant-negative fashion via heterodimerization, allowing a single disabling mutation in one gene to override normal growth controls [37–39]. In addition, the interaction between AURKA and P53 has been reported and discussed [40–43]. Due to the intricate interactions between these two molecules, we were also interested in the role of P53 in our AURKA inhibition scheme and the effect of AURKA inhibition on P53 expression.

Therefore, we hypothesized that the inhibition of AURKA with MLN8237 will decrease survival in lung cancer cell lines and increase sensitivity to irradiation. We first profiled AURKA and P53 expression in NSCLC patient samples and their correlation with survival and then explored the effect of inhibiting AURKA on selected NSCLC cell lines and investigated whether combining AURKA inhibition with radiation therapy is more beneficial than radiation alone in vitro and in vivo. Furthermore, we also examined the necessity of P53 for the mechanism in this system.

Our data demonstrated that AURKA is expressed in most NSCLC samples and is negatively related to overall survival, which is also impacted by P53 expression status. We further showed that MLN8237 could decrease proliferation and increase radiosensitivity of multiple lung cancer cell lines in vitro. This effect was confirmed in vivo using a xenograft mouse model, and the suppression effect might be partially P53 dependent in a cell culture model.

## Methods

### Patient samples

A total of 63 primary lung cancer patients who underwent surgical tumor resection or lobectomy at the Cancer Institute and Hospital of Tianjin Medical University (Tianjin, China) were included in this study. Specimens and clinical information were collected under approval of the University Institutional Review Board. Histological diagnosis was determined by hematoxylin and eosin staining according to the World Health Organization (WHO) classification. Patients’ medical records were reviewed retrospectively and followed up every 3 months until death or until the end of the study (May 2014). The characteristics of these patients are summarized in Additional file 1: Table S1. The statistical relationship between AURKA or other protein expression levels and the clinical characteristics or outcomes, such as stage, lymph node metastasis status, time to progression (TTP), progression-free survival (PFS) and overall survival (OS), were analyzed by two statisticians independently.

### Immunohistochemistry (IHC)

Lung tumor samples were collected, fixed and paraffin embedded within 30 min after surgery. Protein expression levels were evaluated by immunohistochemical staining using the Streptavidin-Peroxidase (SP) Detection IHC kit (Abcam, Cat. ab64264). Antibodies to Ki67, P53 (wild type or mutant) and P21 were ordered from Santa Cruz (Cat. sc-101,861, sc-126, sc-6246), the AURKA antibody was from Abcam (Cat. ab1287), and all were used at a 1:200 dilution. The tumor area of each sample was imaged randomly 30 times under 400-fold magnification and reviewed by two pathologists independently. Using ImageJ software, 100 tumor cells per image were counted and scored as negative (0), 0 points; weakly positive (< 25% positive), 1+; moderately positive (25–50% positive), 2+; or strongly positive (> 50% positive), 3+. Immunohistochemistry results were evaluated by a semiquantitative approach used to assign an H-score to tumor samples, which was calculated as [1 × (% cells 1+) + 2 × (% cells 2+) + 3 × (% cells 3+)]. This process was repeated on the 30 images, and the final score was calculated as the average of the H-scores [44].

### Cell culture and reagents

Human lung cancer cell lines from the American Type Culture Collection (ATCC) with their standard Cell Line Authentication and Characterization (H460, H1299 and A549) were utilized. HCC2429 cells were kindly provided by Dr. Tao Dang (Vanderbilt University, Nashville, TN) [45]. H1299 Tet-ON P53^WT^ cells were a gift from Dr. Steven B. McMahon (Thomas Jefferson University, Philadelphia, PA), and the wildtype P53 expression in these cells can be induced by 500 ng/ml tetracycline/doxycycline for 2–12 h [46]. Cells were subcultured (less than 6 months before being reconstituted from frozen stocks) in RPMI 1640 (Thermo Fisher, Cat. 61,870–036) supplemented with 10% fetal bovine serum (GE Healthcare, Cat. SH30071.03HI), 100 units/ml penicillin, and 100 μg/ml streptomycin (Thermo Fisher, Cat. 10,378,016) at 37 °C in a humidified atmosphere containing 5% CO_2_ and tested for *Mycoplasma* contamination every 2 months during the experiment [47].

### Cell viability assay and clonogenic assay

MLN8237 was kindly provided by Takeda Oncology Inc. (Cambridge, MA). The compound was dissolved in DMSO (Sigma, Cat. D2650) as a stock solution (10 mM) and then diluted freshly to desired concentrations in RPMI 1640 containing serum before cell growth experiments. The effect of MLN8237 on cell viability was analyzed via MTS assay using the CellTiter 96 cell proliferation assay kit (Promega, Cat. G5430). Cells were seeded in 96-well plates at 3000 cells per well and treated with various concentrations of MLN8237 24 h post adhesion. The MTS assay was conducted at 24, 48, and 72 h after treatment. An equivalent amount of DMSO for the highest concentration of drug was used as a vector control. Drug toxicity was compared by normalizing cell survival to the control. Experiments were performed in triplicate. The effect on radiation resistance was measured by colony formation assay. A total of 100–800 cells were seeded into 60-mm cell culture dishes, cultured for 8 h for attachment, and then treated with DMSO (control) or MLN8237 for 2 h at 37 °C post adhesion. After radiation (0, 2, 4, or 6 Gy), cells were incubated at 37 °C with 5% CO_2_ for 10–14 days. Cells were then fixed for 20 min with 70% ethanol and stained for 15 min in 0.5% crystal violet solution (Sigma, Cat. V5265). Colonies, defined as clusters of at least 50 cells, were counted, and the plating efficiency (PE, No. of colonies formed / No. of cells seeded × 100%) and surviving fraction (SF, No. of colonies formed after treatment / No. of cells seeded × PE) were calculated individually. Finally, the dose enhancement ratio (DER) was calculated as the radiation dose that yielded a surviving fraction of 0.2 for vehicle (DMSO)-treated cells divided by that for MLN8237-treated cells after correcting for drug toxicity [48].

### Microscopic observation of cellular morphology

The morphology of the cultured cells was examined regularly using a phase contrast inverted microscope (Olympus IX71). Their shape and appearance were captured, and the essential signs of deterioration were analyzed by ImageJ software, including the length of the cell axis, granularity around the nucleus, detachment of the cells from the substrate, and cytoplasmic vacuolation. Alive epithelial-like cells are polygonal in shape with more regular dimensions and grow attached to a substrate in discrete patches; cells with greatly enlarged cellular size were characterized as senescent cells; and cells undergoing significant size shrinkage and chromatin condensation or cytoplasm vacuolation were quantified as apoptotic cells. Finally, the ratio of cells with different morphological changes was analyzed using statistical software [49].

### Western blot analysis

Cultured cells were lysed in M-PER (Thermo Fisher, Cat. 78,501) protein extraction reagent with protease and phosphatase inhibitor cocktail. Cell lysates were centrifuged at 9000×*g* for 10 min at 4 °C. Supernatants were transferred to clean microcentrifuge tubes, frozen on dry ice, and thawed on ice. Total protein concentrations in the lysates were determined using the Pierce BCA Protein Assay Kit (Thermo Fisher, Cat. 23,250). Equal amounts of total proteins (30 μg/lane unless stated otherwise) were loaded on a 10% SDS-PAGE gel. Membranes were subsequently incubated with various primary antibodies. To investigate P53 signaling, HCC1299 Tet-ON P53^WT^ cells were treated with tetracycline (0.5 μg/mL) 2 h post cell adhesion prior to MLN8237 with or without radiation administration. Cells were harvested 48 h posttreatment, and extracted protein was subjected to immunoblotting as described above. Primary antibodies against P53, P21, caspase 3 and PARP1 were purchased from Santa Cruz (Cat. sc-126, sc-6246, sc-7272, and sc-8007; 1:1000 dilution), and the reference beta-actin was from Sigma (Cat. A2066, 1:8000). Experiments were performed in triplicate.

### Tumor xenograft assay and tumor tissue IHC analysis

All experiments were performed according to protocols approved by the Institutional Animal Care and Use Committee (IACUC) of Thomas Jefferson University and complied with the Guide for the Care and Use of Laboratory Animals. Female 6- to 8-week-old athymic nude mice (Jackson, Cat. 002019) were injected with 3 × 10^5^ H460 cells subcutaneously in the right hind flank. When tumors reached a volume of approximately 50–300 mm^3^ (palpable lesions), mice were assigned to one of the following treatment groups (6 per group, matched tumor size): 1) vehicle control (orally treated with vehicle); 2) MLN8237 (30 mg/kg/d p.o.) for 30 days using a previous protocol [50]; 3) RT group treated with radiation 2 Gy per day for 5 days (2 Gy/f, 5 days); and 4) combination group treated with RT (2 Gy/f, 5 days) and MLN8237 (30 mg/kg/d, p.o. 30 days). The length (L) and width (W) of the subcutaneous tumors were measured by calipers every 3 days, and the tumor volume (V) was calculated as V = (L × W × W)/2. Mice were sacrificed at the end of the study or euthanized if their tumor reached 2000 mm^3^. Tumor sections (end of the treatment) were embedded in paraffin for caspase 3 IHC analysis (Cell Signaling Technology, Cat. 9579S, 1:500 dilution).

### Statistical analysis

The correlation of protein expression with clinical characteristics was assessed by the chi-square test. The survival analysis was performed using the Kaplan-Meier method with log-rank univariate analysis and Cox regression multivariate analysis. Overall survival (OS) is defined as the time between the date of the pathological diagnosis to the date of death or last follow-up. Progression-free survival (PFS) is defined as the time between the pathological diagnosis and the earliest signs of disease progression as determined by CT or MRI imaging using RECIST (Response Evaluation Criteria In Solid Tumors) criteria, death from any cause, or censored at the last check-up date. It was defined as the time to progression (TTP) when deaths were censored (https://www.federalregister.gov/documents/2015/04/22/2015-09303/clinical-trial-endpoints-for-the-approval-of-non-small-cell-lung-cancer-drugs-and-biologics-guidance). All data were analyzed using Prism 7 (GraphPad) and/or SPSS 25 (IBM SPSS Inc.), and *p* < 0.05 was considered statistically significant.

## Results

### AURKA expression correlates with decreased survival in NSCLC

**Fig. 1:**
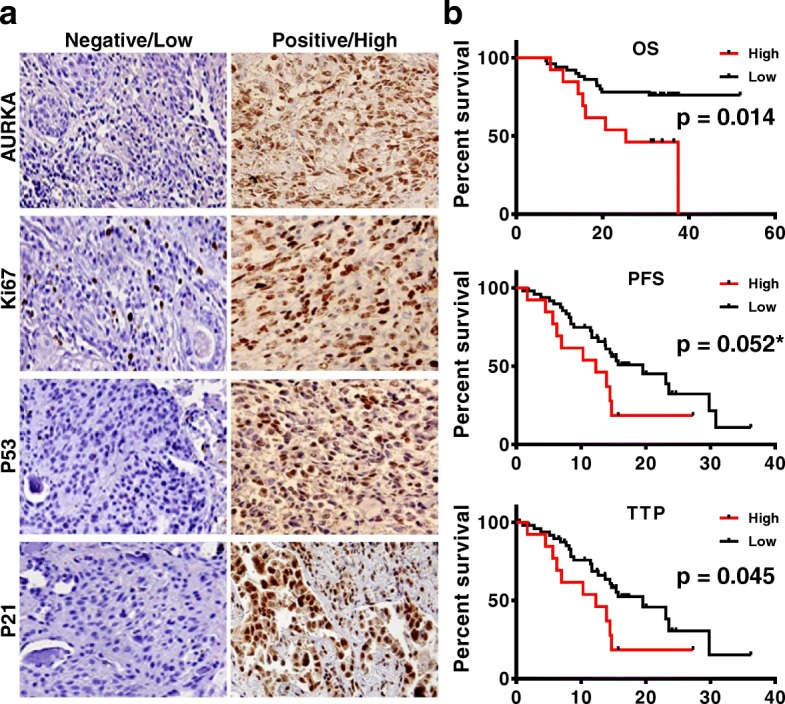
Representative specimens of immunohistochemistry staining and survival analysis of AURKA expression. **a** Negative or low expression (left) vs positive or high expression (right) of AURKA, Ki67, P53 and P21, Magnification 400X. **b** Correlation of Aurora A expression to patient survival. High Aurora A expression is linked to decreased overall, progression free survival and time to progression. Kaplan-Meier survival analysis using Log-rank (Mantel-Cox) test, * *p* = 0.046 if using Gehan-Breslow-Wilcoxon test. *p* < 0.05 means statistical significance

Clinical characteristics of the 63 lung cancer patients, including age, sex, cancer type, stage, lymph node status, and the relationship between AURKA expression, are summarized in Additional file 1: Table S1. The rate of positive staining for AURKA was 93.7% (59/63); of these 59 samples, 22% (13 of the total 59 patients) expressed relatively higher levels of the protein; 78% (46) expressed lower levels. Although most samples have a positive AURKA signal (Fig. 1a), no significant association was found between AURKA expression and other clinicopathologic features, such as tumor type and stage (Additional file 1: Table S1). We then analyzed the expression level of AURKA on patient survival. The Kaplan–Meier analysis indicated that the overall survival (OS) of patients with high AURKA expression was significantly poorer than those with low expression (*p* < 0.05, Fig. 1b). Although progression-free survival (PFS) was only significantly different when using the Gehan-Breslow-Wilcoxon test, not the Log-rank (Mantel-Cox) test (*p* = 0.046 vs. *p* = 0.052, respectively), elevated expression of AURKA predicted an inferior time to progression (TTP) with a median duration of 12.3 vs. 19.6 months, respectively (p < 0.05, Fig. 1b, Table 1). Further analysis was performed to assess Ki67, P53, and P21 expression and other clinical statuses on patient survival. As listed in Table 1, patients with higher expression of Ki67, positive lymph node metastasis, or higher grade tumors have significantly shorter survival times or TTP. Although the expression level of P53 or P21 was not related to patient survival, multivariate survival analysis using Cox’s regression model showed that the OS was the shortest when total AURKA and P53 were overexpressed, while the longest survival time occurred when both of their expression levels were lower or negative. Moreover, the survival curve fell in the middle when either one was highly expressed (Table 2). This analysis showed that AURKA is expressed in most NSCLCs and might be a predictive factor of poor prognosis and that P53 status might also influence overall survival.

**Table 1:** univariate analysis for overall, progression free survival and time to progression

		Overall Survival				Progression Free Survival				Time to Progression			
	Total cases	Died *n* = 20	Alive *n* = 43	Log-rank	Gehan test	Prog. *n* = 37	Prog. free *n* = 26	Log-rank	Gehan test	Prog. *n* = 34	Prog. free *n* = 29	Log-rank	Gehan test
Age (years)													
≥ 57	32	11 (55)	21 (49)	0.806	0.748	21 (57)	11 (42)	0.5057	0.2854	19 (56)	13 (45)	0.664	0.302
< 57	31	9 (45)	22 (51)			16 (43)	15 (58)			15 (44)	16 (55)		
Gender, n (%)													
Female	16	7 (35)	9 (20)	0.358	0.643	11 (30)	5 (19)	0.405	0.4016	10 (29)	6 (21)	0.499	0.424
Male	47	13 (65)	34 (79)			26 (70)	21 (71)			24 (74)	23 (79)		
Pathology, n (%)													
SCC	32	10 (50)	22 (51)	0.982	0.953	19 (51)	13 (50)	0.832	0.663	17 (50)	15 (52)	0.928	0.596
ADC	31	10 (50)	21 (49)			18 (49)	13 (50)			17 (50)	14 (48)		
Lymph node metastasis, n (%)													
Pos	29	15(75)	14 (33)	0.004**	0.008**	24 (65)	5 (19)	0.006*	0.032*	22 (65)	7 (24)	0.002**	0.015*
Neg	34	5 (25)	29 (67)			13 (35)	21 (71)			12 (35)	22 (76)		
Stage, n (%)													
IA-IB	20	2 (10)	18 (42)	0.049*	0.049*	7 (19)	13 (50)	0.001**	0.006**	7 (21)	13 (45)	0.000***	0.005**
IIA-IIB	14	5 (25)	9 (21)			6 (16)	8 (31)			5 (15)	9 (31)		
IIIA-IIIB	26	11 (55)	15 (35)			21 (57)	5 (19)			19 (56)	7 (24)		
IV	3	2 (10)	1 (2)			3 (8)	0 (0)			3 (9)	0 (0)		
Ki67, n (%)													
High	39	18 (90)	21 (49)	0.001**	0.002**	27 (73)	12 (46)	0.004**	0.006**	25 (74)	14 (48)	0.014*	0.012*
Low	24	2 (10)	22 (51)			10 (27)	14 (54)			9 (26)	15 (52)		
AURKA, n (%)													
High	13	8 (40)	5 (12)	0.010*	0.013*	10 (27)	3 (12)	0.052	0.046*	10 (29)	3 (10)	0.045*	0.037*
Low	50	12 (60)	38 (88)			23 (73)	23 (88)			24 (71)	26 (90)		
P53, n (%)													
High	27	11 (55)	16 (37)	0.154	0.107	15 (41)	14 (54)	0.439	0.562	13 (38)	14 (48)	0.669	0.785
Low	36	9 (45)	27 (63)			22 (59)	12 (46)			21 (62)	15 (52)		
P21, n (%)													
High	20	8 (40)	12 (28)	0.266	0.296	11 (30)	9 (35)	0.905	0.898	9 (26)	11 (38)	0.696	0.733
Low	43	12 (60)	31 (72)			26 (70)	17 (65)			25 (74)	18 (62)		
P53 + AURKA, n (%)													
DP	7	4 (20)	3 (7)	0.042*	0.012*	5 (13)	2 (8)	0.767	0.527	5 (15)	2 (7)	0.829	0.594
SP	26	11 (55)	15 (35)			15 (41)	11 (42)			13 (38)	13 (45)		
DN	30	5 (25)	25 (58)			17 (46)	13 (50)			16 (47)	14 (48)		

*Log-rank* Log-rank (Mantel-Cox) test, *Gehan test* Gehan-Breslow-Wilcoxon test, *DP* Double positive, *SP* Single positive, *DN* Double negative; *p < 0.05, ** *p* < 0.01, ***p < 0.0001, negative expression cases were included in the Low expression group

**Table 2:** Multivariate Cox proportional-hazards analysis for overall and progression free survival

	Covariate	b	SE	Wald	P	Exp(b)	95% CI of Exp(b)
Overall Survival	AURKA+P53	0.7543	0.31	5.935	0.015	2.1262	1.1588 to 3.9009
	Ki67	1.6499	0.629	6.878	0.009	5.207	1.5171 to 17.8679
	Stage	0.6581	0.276	5.673	0.017	1.931	1.1236 to 3.3192
	LN	1.3798	0.519	7.073	0.008	3.974	1.4375 to 10.9865
Progression Free Survival	Ki67	0.7902	0.364	4.725	0.030	2.204	1.0808 to 4.4936
	Stage	0.6014	0.22	7.447	0.006	1.825	1.1847 to 2.8105
	LN	1.0779	0.366	8.67	0.003	2.939	1.4339 to 6.0221

*LN* Lymph node metastasis status, data was analyzed by SPSS 25 software, negative expression cases were included in the Low expression group

### MLN8237 inhibits human lung cancer cell viability in vitro

**Fig. 2:**
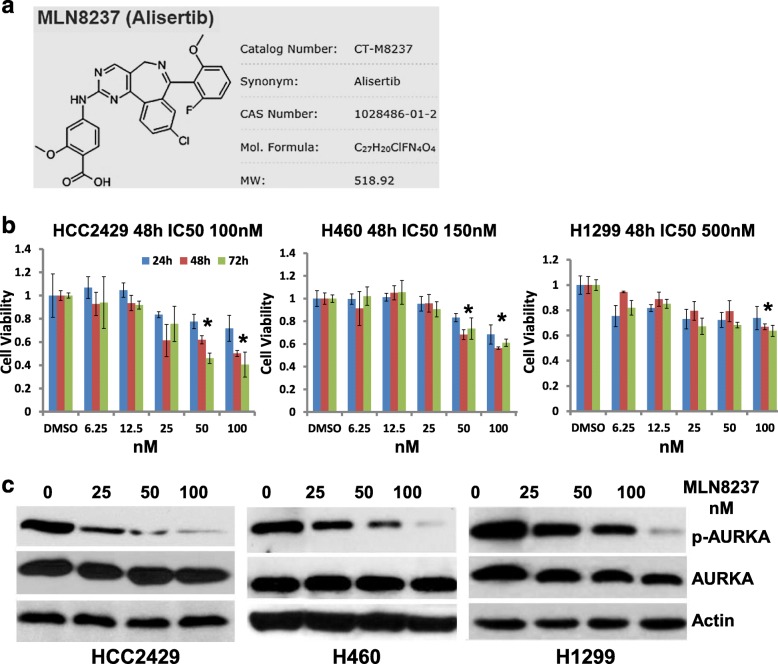
MLN8237 inhibits the growth of human cancer cell lines. **a** Chemical structure and molecular weight of MLN8237 (Alisertib). **b** Effect of MLN8237 on p53-competent (H460 and H2429) and p53-deficient (H1299) human lung cancer cell lines. Cells were treated with the indicated concentrations of MLN8237 for 3 days, and the number of metabolically active viable cells per well was measured by MTS assay. Data are mean ± SD of triplicate wells. At 48 h after 100 nM MLN8237 treatment, cell survival of H460 dropped to 56%, HCC2429 to 50%, and H1299 to 77% compared to a DMSO-treated control. * *p* < 0.05, t-test, groups of 48 h and 72 h. **c** Dose dependent inhibition of AURKA activation by MLN8237. At 48 h after 100 nM MLN8237 treatment, phosphorylation of AURKA was decreased significantly in all cell lines

To investigate the effect of AURKA inhibition on cell proliferation, cell survival after MLN8237 treatment was analyzed in three selected cultured human lung cancer cell lines, the P53-competent cell lines H460 and HCC2429, and the P53-deficient cell line H1299. Cultured cells were treated with multiple concentrations (0–500 nM) of MLN8237 for 24, 48 and 72 h to determine the optimal treatment conditions that would lead to effective suppression of AURKA activation (phospho-AURKA) and cell survival. As shown in Fig. 2b, the survival of three lung cell cancer lines was decreased in a dose-dependent and time-dependent manner. The IC50 values for the HCC2429, H460 and H1299 cells were approximately 100 nM, 150 nM, and 500 nM, respectively. Accordingly, activation of AURKA was inhibited by MLN8237 dose-dependently, and 100 nM is an adequate dose to inhibit phosphorylation of AURKA but not enough for growth suppression in H1299 (Fig. 2c). These survival data showed that cell growth of the P53-competent cells H460 and HCC2429 was sensitive to MLN8237, but the P53-deficient cell line H1299 was not, although all of them were sensitive to MLN8237-induced AURKA inhibition.

### AURKA inhibition via MLN8237 leads to cell morphological changes

**Fig. 3:**
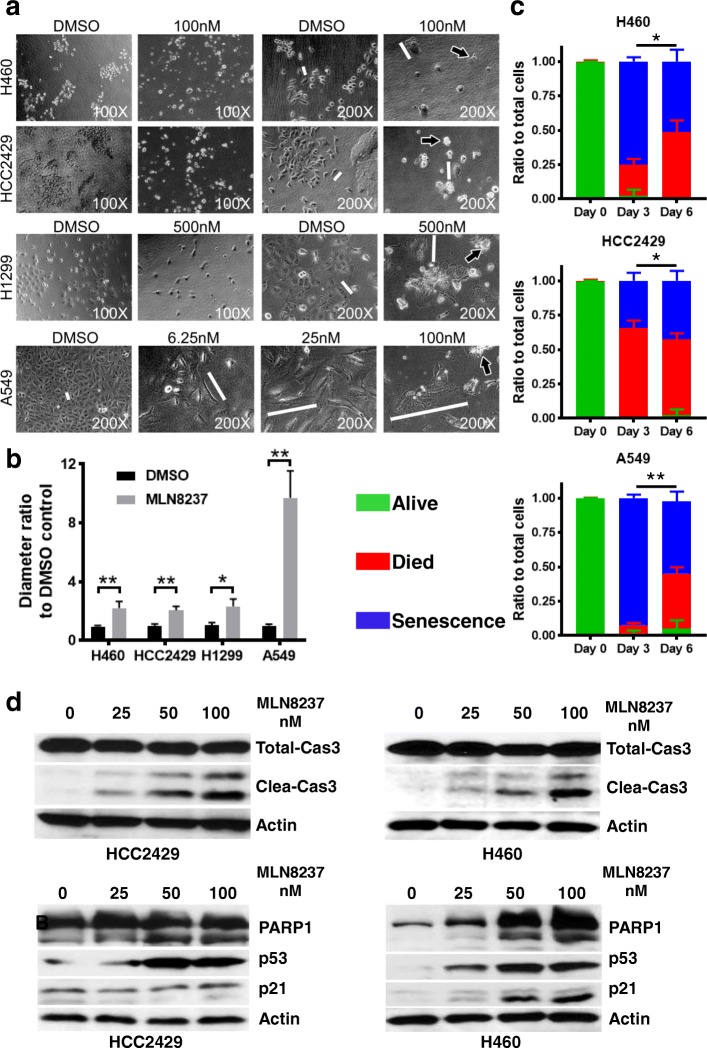
MLN8237 induces programmed cell death and cell senescence. **a** Morphological change after MLN8237 treatment. The length of white bar represents the major axis of cancer cell. The dying cell was marked by arrow filled with black. A549 cell was using as representative cell senescence after treatment. **b** Feret’s diameter ratio of cells shown characteristics of cell senescence. Cell Feret’s diameter was calculated by ImageJ and averaged from 6 cells per image. Bar means mean ± SD, * *p* < 0.05, ** *p* < 0.01. **c** Cell fates distribution after MLN8237 treatment. Green bar means portion of living cells, red bar means portion of dead cells, blue means portion of senescent cells, Data showed as mean ± SD, * *p* < 0.05, ** *p* < 0.01. **d** MLN8237 induces apoptosis and DNA damage signaling in NSCLC cell lines. Cells were treated with increasing concentrations of MLN8237 for 48 h. Caspase-3 antibody recognizes both the full-length and cleaved fragment, PARP1 recognizes both the full-length and cleaved fragments

Previous studies by others have shown that AURKA kinase inhibition leads to cell apoptosis, death and senescence [51, 52]. After 72 h of MLN8237 treatment, microscopic analysis indicated that MLN8237 was sufficient to induce cytostatic activity in the majority of cells. Although cells remained viable, the phenotype was characterized by enlarged cellular size and nucleus along with enlarged cytoplasmic vacuoles and increased levels of lamellipodia and filopodia [49, 53, 54]. There was a clear dose-dependent increase in the major axis of A549 cells, which express wild-type P53 and are sensitive to drug-induced senescence (Fig. 3a, b). As compared in Fig. 3c, the percentage of cell changes was determined by the degree of cell enlargement, which might represent senescence, shrinkage, condensation, and the disintegration of the cell membrane to form apoptotic bodies, or dead cells floating in the media. H460 and A549 cells had more senescent cells after 3 days of MLN8237 treatment, and the prolongation of treatment resulted in more cell death or apoptosis. These data suggested that lung cancer cells were more susceptible to MLN8237-induced senescence than cell death as an early treatment response, although β-galactosidase staining would be a better biomarker.

### Cell death and DNA damage signaling post MLN8237 treatment

To determine whether AURKA inhibition induces cell death and/or senescence signaling, biochemical markers for apoptosis and DNA damage signaling were evaluated by western blot. As shown in Fig. 3d, MLN8237 treatment induced cleavage of caspase 3 and PARP1 in HCC2429 and H460 cells in a dose-dependent manner, which represents the signaling involved in programmed cell death. The upregulated P53 and P21 might also represent DNA damage responses activated in cells undergoing senescence because the tumor suppressors P53 and P21 are particularly important in regulating cellular senescence [55, 56]. These data, combined with the above morphological results, indicated that cell senescence and apoptosis should both be counted in the cellular outcomes associated with AURKA inhibition.

### MLN8237 sensitizes selected lung cancer cell lines to ionizing radiation in vitro

**Fig. 4:**
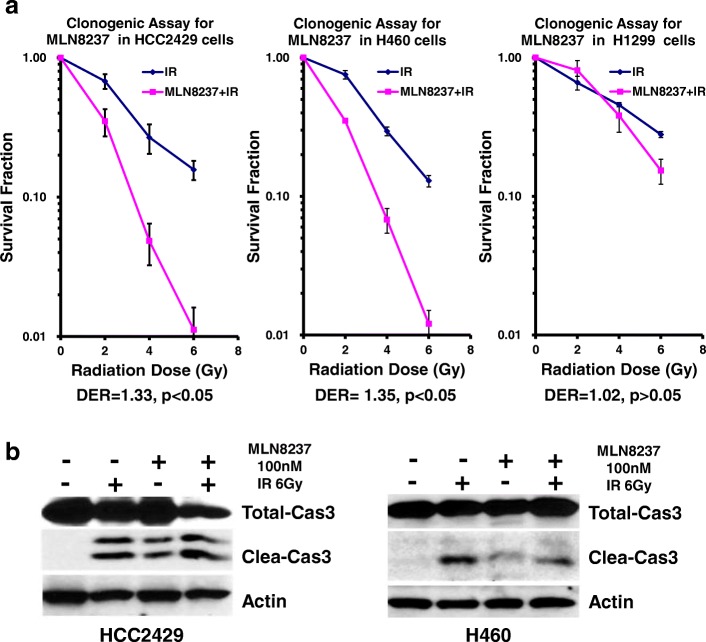
Irradiation enhancement of MLN8237 in p53 expressing NSCLC cell lines. **a** Radio-sensitization of lung cancer cells by MLN8237. HCC2429 (Left), H460 (Middle) and H1299 (right) cells were treated with 100 nM MLN8237 or DMSO for 2 h followed by irradiation with the indicated doses. Forty-eight hours after radiation, drug-containing media was replaced with fresh media. After 8–12 days, surviving colonies were stained and scored. Shown are survival curves containing the mean ± SD of three separate, repeated experiments. **b** Caspase 3 cleavage after MLN8237, radiation or combination treatment

To investigate whether AURKA inhibition radiosensitizes lung cancer cells, clonogenic assays were performed using 0–6 Gy doses of radiation with or without 100 nM MLN8237 treatment. Cell colonies were quantified to generate survival curves as shown in Fig. 4a. After treatment, HCC2429 and H460 cells had increased sensitivity to the lethal effect of radiation, with DERs of 1.33 (*p* < 0.05) and 1.35 (*p* < 0.05), respectively. There was no significant enhancement in the naturally P53-deficient and radiation-resistant H1299 cells with a DER of 1.02 (*p* > 0.05). We then analyzed the effect of combining AURKA inhibition and radiation on caspase 3 cleavage. As shown in Fig. 4b, there were no marked changes in total caspase 3 expression in H460 cells in each group, while combination treatment greatly decreased total caspase 3 expression in HCC2429 cells. Radiation alone can induce stronger caspase 3 cleavage than MLN8237 alone, and the combination has the strongest induction in both cell lines. However, no caspase cleavage was observed in P53-deficient H1299 cells. These data suggest that lower doses of radiation could achieve an equivalent antitumor effect when MLN8237 treatment is combined with radiation compared with radiation alone in vitro, particularly in P53-competent cells.

### Combined radiation and AURKA targeting results in superior tumor inhibition effects in vivo

**Fig. 5:**
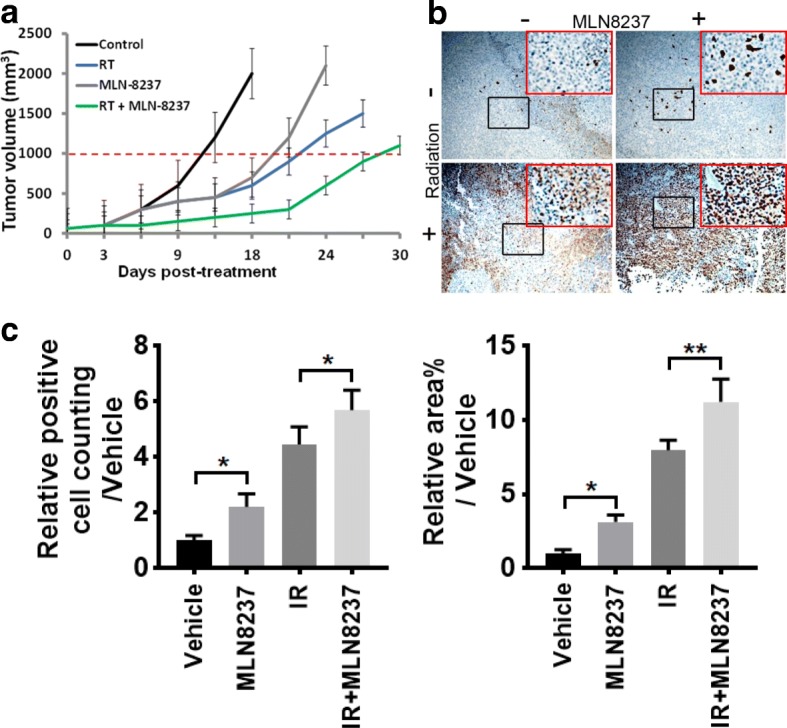
MLN8237 combined with radiation treatment suppress tumor growth in vivo. **a** Combination of MLN8237 and radiation prolongs tumor growth delay in H460 xenograft model. After 6 to 8 days cell injection, mice have palpable subcutaneous tumor were treated with vehicle control, MLN8237 (30 mg/kg for 30 days), radiotherapy (2 Gy daily for 5 consecutive days), or combined MLN8237 and radiotherapy (mice were irradiated 1 h after MLN8237 treatment with 2 Gy daily for 5 consecutive days). Tumor growth delay as defined by the number of days required to reach a tumor volume of 1000 mm^3^ was measured. **b** Representative images of IHC staining for cleaved caspase 3. The magnification of the black box is 200X, the red box is magnified field of the black box (400X). **c** Staining for active caspase 3 was performed to measure apoptosis and the number of positive cells was scored and graphed by averaging three repeated experiments; *p < 0.05, ** *p* < 0.01

To further verify the radio-sensitizing effects of MLN8237 on lung cancer cells in vivo, tumor growth was assessed in a mouse xenograft model using H460 cells. Tumor growth delay was calculated as the number of days required to reach a tumor volume of 1000 mm^3^ for the treatment groups compared with the control group. As Fig. 5a shows, treatment with either MLN8237 or RT significantly delayed the growth of H460-derived tumors by approximately 9 and 11 days, respectively, and the combination group had the greatest tumor growth delay (~ 17 days). We also compared caspase 3 cleavage in tumor sections using IHC. As shown in Fig. 5b, the relative number of positive staining for cleaved caspase 3 of MLN8237 or radiation single treatment was 2.21-fold (*p* < 0.05) and 4.46-fold (*p* < 0.0001) higher than that in the vehicle control group, respectively, and the area % of staining was 3.13-fold (*p* < 0.05) and 7.97-fold (*p* < 0.0001) higher than that in the vehicle control group. The number of positive cleaved caspase 3 staining and the area % of the combination treatment was approximately 1.3- to 4.5-fold higher than those in the radiation or MLN8237 single treatment group (*p* < 0.05), which represents massive apoptotic cell death compared with other groups (Fig. 5c, d). These data confirmed that AURKA inhibition could effectively enhance the radiation efficacy in vivo.

### Survival inhibition and the radio-sensitizing effects of MLN8237 might be influenced by P53 expression

**Fig. 6:**
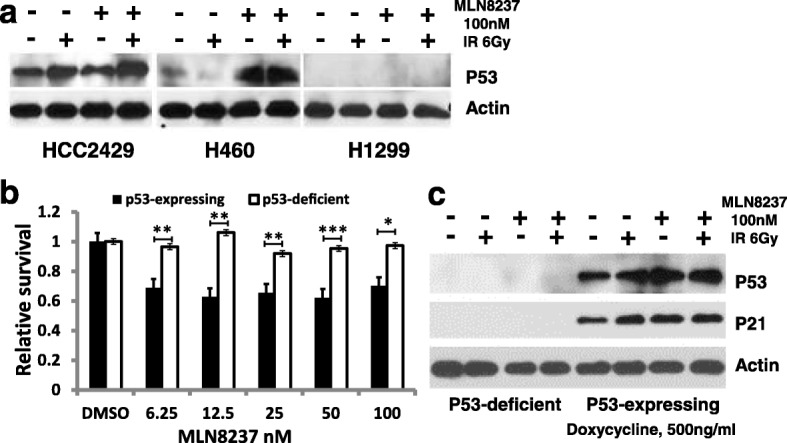
Restored P53 function reverse MLN8237 resistance. **a** P53 induction in H460 and HCC2429 cells post 48 h MLN8237, radiation or combined treatment. **b** Cell survival after MLN8237 treatment when P53 was induced in H1299 cells by Doxycycline. T-test, ** *p* < 0.01. **c** DNA damage signaling after MLN8237, radiation, and combined treatment in H1299 cells with restored P53 function. Markable P53 and P21 can be induced by AURKA inhibition or radiation alone or combined

The above data showed that the P53-deficient lung cancer cells H1299 are resistant to MLN8237-induced cell death or radiation enhancement, although AURKA activation could be suppressed at the same concentration in P53-competent HCC2429 and H460 cells. Thus, we first assessed P53 levels after treatment in these cells. Western blot analysis showed that MLN8237 treatment could induce notable increased P53 expression in HCC2429 and H460 cells, while radiation alone could induce increased P53 expression in HCC2429 cells. The strongest induction was observed after combination treatment in HCC2429 and H460 cells. There was no P53 expression in any treatment group of H1299 cells (Fig. 6a). Thus, to investigate whether restoration of P53 function in P53-deficient H1299 cells would enhance growth suppression by MLN8237, a Tet-On H1299 cell line with an inducible wild-type P53 expression system was used for the mechanism study. Compared with parental cells without P53 expression, MLN8237 could suppress cell growth significantly after induced P53 expression in H1299 cells with doxycycline. All MLN8237 treatment groups, even at the lowest concentration (6.25 nM), had approximately 30% (*p* < 0.01) increased inhibition of cell survival (Fig. 6b). We further investigated DNA damage signaling after combination treatment. Western blot analysis showed that MLN8237 or radiation treatment increased P53 and P21 expression only in the natively P53-expressing cell lines H460 and HCC2429 without doxycycline induction (Figs. 3, 6a). With doxycycline induction, notable P53 and P21 expression was found and further induced by MLN8237 or radiation alone treatment, and the highest expression of P53 and P21 was observed in the combination treatment group (Fig. 6c). All of these data suggest that cell growth suppression after AURKA inhibition might be partially P53 dependent in selected NSCLC cell lines in vitro.

## Discussion

AURKA plays an important role during mitosis, including centrosome separation, spindle assembly, chromosome segregation, and cytokinesis [11, 12]. Overexpression of AURKA has been linked to the development of some types of tumors [13], and several studies have correlated its expression to poor differentiation [14–16], high tumor aggressiveness, and lymph node metastasis [18, 30]. It has also been linked to chemoresistance [17, 18], and its inhibition has been suggested as a mechanism of radiosensitization [27–29]. This manuscript details the effects of inhibiting AURKA phosphorylation via MLN8237 (Alisertib) on the growth and radiosensitivity of selected NSCLC cell lines and further investigated the mechanism.

First, we analyzed 63 NSCLC patient samples to determine the potential clinical impact of AURKA expression. Most of the analyzed samples stained positive for AURKA (Table 1), and the increased expression correlated with the decreased overall and progression-free survival very well (Fig. 1b), which is consistent with previous studies in breast [57, 58], gastric [59], ovarian [60], colon [61], and lung [62, 63] cancers. It should be noted that P53 expression status might influence the prognostic potential of AURKA in NSCLC, as our data showed that P53 and AURKA were jointly related to overall survival (Fig. 1b, Table 2). Although the difference in progression-free survival was not statistically significant, for the TTP, it was significantly related. Further investigation might help to solve the puzzle of whether we can identify if the detected overexpression of P53 in patient tissues is associated with a mutant or wild-type p53 gene.

Serial publications have already confirmed that Aurora kinase inhibition can induce programmed cell death and cellular senescence in vitro and in vivo [51, 52, 64, 65]. Because typical morphological changes in apoptotic and dead cells are a rounded shape, cell shrinkage and lost adhesion to the flask surface, and even floating fragments, whereas senescent cells have flattened and enlarged cell shapes [53], we further explored the effects of AURKA inhibition by assessing the cell survival, cell morphology changes and effect on radiosensitivity in three NSCLC cell lines. After targeting AURKA by MLN8237, the survival of H460, HCC2429, and H1299 cells was decreased in a dose-dependent manner (Fig. 2b), and the sensitivity could be ranked as HCC2429 > H460 > H1299 according to their IC50, although all their AURKA activities could be inhibited at the same low concentration (Fig. 2c). Programed cell death and senescence were major death pathways because of the induced expression of P53 and P21 (Fig. 3), which was consistent with reports of AURKA suppression resulting in cell senescence and apoptosis [51–53]. Hence, it might also explain why H1299 cells were resistant to AURKA inhibition in part because of their natural P53 deficiency. It should be noted that H1299 cells showed cellular senescence and cell survival inhibition (50%) at very high concentrations of MLN8237 (500 nM). Other mechanisms independent of P53 expression might contribute as well through AURKA and other signaling pathways. For example, MA et al. found that tanshinones could induce miR-32 expression, which can target and suppress downstream AURKA and result in cell apoptosis in H1299 [66].

It is well known that ionizing radiation induces DNA damage signaling and causes P53-dependent apoptosis [36–38]. Therefore, we expected that decreasing AURKA phosphorylation and the subsequent induced P53 expression or activation would lead to an increased susceptibility to irradiation. Indeed, the clonogenic assay confirmed that the combination of MLN8237 and radiation treatment significantly enhanced radiation sensitivity in P53-expressing cells (H460 and HCC2429) at the low dose of 2 Gy but not in the P53-deficient H1299 cell line (Fig. 4a). Moreover, the data from the H460 xenograft experiment demonstrated that the combination treatment had the strongest tumor growth delay and induced cell death effects compared with the single treatment (Fig. 5), which indicated that AURKA target inhibition could suppress tumor growth and enhance irradiation sensitivity in vivo in the P53-competent cells*.*

The tumor suppressor P53 plays many roles in tumor development, including the regulation of cell cycle progression, induction of apoptosis, and inhibition of cancer genes [39]. Loss of mutant P53 protein and inhibition of the function of wild-type P53 protein are related to the growth of tumors, poor prognosis, and resistance to chemotherapy and radiation therapy [46, 67]. Recent studies have shown that the AURKA and P53 pathways are linked via inter-regulation [21, 42]. Together with these reports and all of our clinicopathologic, in vitro and in vivo data, it suggests that P53 might be an important modulator for AURKA targeting. Therefore, we used a modified H1299 cell line that is capable of expressing wild-type P53 when induced by doxycycline to investigate the mechanism of action. Compared with parental P53-deficient H1299, restored P53 expression in the H1299 Tet-On cells resulted in dramatic cell growth inhibition after AURKA inhibition (Fig. 6b) and induced DNA damage signaling when combined with radiation (Fig. 6c).

Because AURKA can inhibit the activity of P53 in a variety of ways, such as increasing MDM2-mediated degradation of P53 or decreasing binding capability [21, 24], inhibiting AURKA phosphorylation could reactivate P53 [41]. Thus, increased P53 and downstream signaling activity might, in turn, result in cell cycle arrest and programmed cell death or senescence in P53-competent cells. A lower dose of MLN8237 could not induce this type of inhibition in P53-deficient cells, while restored P53 function in this deficient cell could rescue the cell growth suppression and the radiation-sensitizing effect. These results are consistent with previously published reports that P53 is inhibited by AURKA and might imply that AURKA inhibition is partially P53 dependent, at least in our system.

However, there must be other mechanisms at work as well because both P53-expressing cell lines actually showed more or the same caspase 3 cleavage by irradiation alone than by either MLN8237 or combination treatment. This observation agrees with previously published data. Kojima found that inactivating Aurora kinases induces apoptosis in AML cell lines, but this effect was abrogated when P53 was also blocked [68]. Dar has suggested that in the absence of P53, P73 can compensate and result in cell death [23].

## Conclusion

Our data demonstrated that AURKA was commonly expressed in the investigated NSCLC samples and was associated with a poor prognosis, and the expression of P53 might contribute to overall survival in association with AURKA. MLN8237 could significantly inhibit cell proliferation and enhance the radiosensitivity of NSCLC cells in vitro and in vivo through induced programmed cell death and cell senescence by targeting AURKA*,* and these effects were P53-dependent in part because the P53-deficient lung cancer cell line was resistant to AURKA inhibition-induced antiproliferative effects. Although the clinical trials of MLN8237 have been halted [69–72], our findings shed new light on this drug category for NSCLC treatment in that relative expression of AURKA and P53 may be of prognostic value and warrants further investigation with larger, prospective studies. Taken together, this work suggested that pharmacological inhibition of AURKA is a potentially promising therapy when combined with radiotherapy for patients with P53 expression.

## Supplementary information

**Additional file 1: Table S1.** Association of AURKA expression with patient’s clinicopathologic characteristics in NSCLC.

## Data Availability

All data generated or analyzed during this study are included in this published article, and freely available to scientists.

## Abbreviations

ATCC: American Type Culture Collection
AURKA: Aurora kinase A
E2F3: E2F Transcription Factor 3
FBXW7: F-Box And WD Repeat Domain Containing 7
IACUC: Institutional Animal Care and Use Committee
IHC: Immunohistochemistry
NSCLC: Non-small cell lung cancer
OS: Overall survival
PFS: Progression-free survival
PTEN: Phosphatase And Tensin Homolog
RECIST: Response Evaluation Criteria In Solid Tumors
RT: Radiation therapy
SP: Streptavidin-Peroxidase
TTP: Time to progression
WHO: World Health Organization

## References

[1] Bernard WS, Paul K. World Cancer Report 2014: IARC Publications; 2013. http://publications.iarc.fr/Non-Series-Publications/World-Cancer-Reports/World-Cancer-Report-2014

[2] Siegel RL, Miller KD, Jemal A (2018). Cancer statistics, 2018. CA Cancer J Clin.

[3] Nadkar A, Pungaliya C, Drake K, Zajac E, Singhal SS, Awasthi S (2006). Therapeutic resistance in lung cancer. Expert Opin Drug Metab Toxicol.

[4] Provencio M, Sanchez A, Garrido P, Valcarcel F (2010). New molecular targeted therapies integrated with radiation therapy in lung Cancer. Clin Lung Cancer.

[5] Overgaard J (2007). Hypoxic radiosensitization: adored and ignored. J Clin Oncol.

[6] Morgan MA, Lawrence TS (2015). Molecular pathways: overcoming radiation resistance by targeting DNA damage response pathways. Clin Cancer Res.

[7] Rodríguez-Ruiz ME, Vanpouille-Box C, Melero I, Formenti SC, Demaria S (2018). Immunological mechanisms responsible for radiation-induced Abscopal effect. Trends Immunol.

[8] Hufnagl A, Herr L, Friedrich T, Durante M, Taucher-Scholz G, Scholz M (2015). The link between cell-cycle dependent radiosensitivity and repair pathways: a model based on the local, sister-chromatid conformation dependent switch between NHEJ and HR. DNA Repair (Amst).

[9] Bristow RG, Alexander B, Baumann M, Bratman SV, Brown JM, Camphausen K (2018). Combining precision radiotherapy with molecular targeting and immunomodulatory agents: a guideline by the American Society for Radiation Oncology. Lancet Oncol..

[10] Hochegger H, Hegarat N, Pereira-Leal JB (2013). Aurora at the pole and equator: overlapping functions of Aurora kinases in the mitotic spindle. Open Biol.

[11] Wang Y, Sun H, Wang Z, Liu M, Qi Z, Meng J (2014). Aurora-A: a potential DNA repair modulator. Tumour Biol.

[12] Sen S, Katayama H, Sasai K (2008). Functional significance of Aurora kinase a in centrosome amplification and genomic instability. Adv Exp Med Biol.

[13] Fu J, Bian M, Jiang Q, Zhang C (2007). Roles of Aurora kinases in mitosis and tumorigenesis. Mol Cancer Res.

[14] Schneider MA, Christopoulos P, Muley T, Warth A, Klingmueller U, Thomas M (2017). AURKA, DLGAP5, TPX2, KIF11and CKAP5: five specific mitosis-associated genes correlate with poor prognosis for non-small cell lung cancer patients. Int J Oncol.

[15] Ramani P, Nash R, Rogers CA (2015). Aurora kinase a is superior to Ki67 as a prognostic indicator of survival in neuroblastoma. Histopathology..

[16] Zhang J, Li B, Yang Q, Zhang P, Wang H (2015). Prognostic value of Aurora kinase a (AURKA) expression among solid tumor patients: a systematic review and meta-analysis. Jpn J Clin Oncol.

[17] Mignogna C, Staropoli N, Botta C, De Marco C, Rizzuto A, Morelli M (2016). Aurora kinase a expression predicts platinum-resistance and adverse outcome in high-grade serous ovarian carcinoma patients. J Ovarian Res.

[18] Xu J, Yue CF, Zhou WH, Qian YM, Zhang Y, Wang SW (2014). Aurora-a contributes to cisplatin resistance and lymphatic metastasis in non-small cell lung cancer and predicts poor prognosis. J Transl Med.

[19] De Luca M, Lavia P, Guarguaglini G (2006). A functional interplay between Aurora-a, Plk1 and TPX2 at spindle poles: Plk1 controls centrosomal localization of Aurora-a and TPX2 spindle association. Cell Cycle.

[20] Nikonova AS, Astsaturov I, Serebriiskii IG, Dunbrack RL, Golemis EA (2013). Aurora a kinase (AURKA) in normal and pathological cell division. Cell Mol Life Sci.

[21] Katayama H, Sasai K, Kawai H, Yuan ZM, Bondaruk J, Suzuki F (2004). Phosphorylation by aurora kinase a induces Mdm2- mediated destabilization and inhibition of p53. Nat Genet.

[22] Maxwell CA, Benítez J, Gómez-Baldó L, Osorio A, Bonifaci N, Fernández-Ramires R (2011). Interplay between BRCA1 and RHAMM regulates epithelial apicobasal polarization and may influence risk of breast cancer. PLoS Biol.

[23] Dar AA, Belkhiri A, Ecsedy J, Zaika A, El-Rifai W (2008). Aurora kinase a inhibition leads to p73-dependent apoptosis in p53-deficient cancer cells. Cancer Res.

[24] Liu Q, Kaneko S, Yang L, Feldman RI, Nicosia SV, Chen J (2004). Aurora-a abrogation of p53 DNA binding and transactivation activity by phosphorylation of serine 215. J Biol Chem.

[25] Wu CC, Yang TY, Yu CT, Phan L, Ivan C, Sood AK (2012). p53 negatively regulates Aurora a via both transcriptional and posttranslational regulation. Cell Cycle.

[26] Tagal V, Wei S, Zhang W, Brekken RA, Posner BA, Peyton M (2017). SMARCA4-inactivating mutations increase sensitivity to Aurora kinase a inhibitor VX-680 in non-small cell lung cancers. Nat Commun.

[27] Woo JK, Kang JH, Shin D, Park SH, Kang K, Nho CW (2015). Daurinol enhances the efficacy of radiotherapy in lung Cancer via suppression of Aurora kinase a/B expression. Mol Cancer Ther.

[28] Bhatia S, Hirsch K, Sharma J, Oweida A, Griego A, Keysar S (2016). Enhancing radiosensitization in EphB4 receptor-expressing head and neck squamous cell carcinomas. Sci Rep.

[29] Li N, Maly DJ, Chanthery YH, Sirkis DW, Nakamura JL, Berger MS (2015). Radiotherapy followed by aurora kinase inhibition targets tumor-propagating cells in human glioblastoma. Mol Cancer Ther.

[30] Pezzani R, Rubin B, Bertazza L, Redaelli M, Barollo S, Monticelli H (2016). The aurora kinase inhibitor VX-680 shows anti-cancer effects in primary metastatic cells and the SW13 cell line. Investig New Drugs.

[31] Borisa AC, Bhatt HG (2017). A comprehensive review on Aurora kinase: small molecule inhibitors and clinical trial studies. Eur J Med Chem.

[32] Gautschi O, Heighway K, Mack PC, Purnell PR, Lara PN, Gandara DR (2008). Aurora kinases as anticancer drug targets. Clin Cancer Res.

[33] Claire Dees E, Cohen RB, von Mehren M, Stinchcombe TE, Liu H, Venkatakrishnan K (2012). Phase I study of aurora A kniase inhibitor MLN8237 in advanced solid tumors: safety, pharmacokinetics, pharmacodynamics, and bioavailability of two oral formulations. Clin Cancer Res.

[34] Melichar B, Adenis A, Lockhart AC, Bennouna J, Dees EC, Kayaleh O (2015). Safety and activity of alisertib, an investigational aurora kinase a inhibitor, in patients with breast cancer, small-cell lung cancer, non-small-cell lung cancer, head and neck squamous-cell carcinoma, and gastro-oesophageal adenocarcinoma: a five-arm phase 2 study. Lancet Oncol.

[35] Falchook G, Kurzrock R, Gouw L, Hong D, McGregor KA, Zhou X (2014). Investigational Aurora a kinase inhibitor alisertib (MLN8237) as an enteric-coated tablet formulation in non-hematologic malignancies: phase 1 dose-escalation study. Investig New Drugs.

[36] Cuddihy AR, Jalali D, Coackley C, Bristow RG (2008). WTp53 induction does not override MTp53 chemoresistance and radioresistance due to gain-of-function in lung cancer cells. Mol Cancer Ther.

[37] Lee JM, Bernstein L (1993). p53 mutations increase resistance to ionizing radiation. Proc Natl Acad Sci.

[38] Bristow RG, Brail L, Jang A, Peacock J, Chung S, Benchimol S (1996). p53-mediated radioresistance does not correlate with metastatic potential in tumorigenic rat embryo cell lines following oncogene transfection. Int J Radiat Oncol Biol Phys.

[39] Sigal A, Rotter V (2000). Oncogenic mutations of the p53 tumor suppressor: the demons of the guardian of the genome. Cancer Res.

[40] Katayama H, Wang J, Treekitkarnmongkol W, Kawai H, Sasai K, Zhang H (2012). Aurora kinase-A inactivates DNA damage-induced apoptosis and spindle assembly checkpoint response functions of p73. Cancer Cell.

[41] Mao J-H, Wu D, Perez-Losada J, Jiang T, Li Q, Neve RM (2007). Crosstalk between Aurora-a and p53: frequent deletion or downregulation of Aurora-a in tumors from p53 null mice. Cancer Cell.

[42] Hsueh KW, Fu SL, Chang CB, Chang YL, Lin CH (1834). A novel Aurora-A-mediated phosphorylation of p53 inhibits its interaction with MDM2. Biochim Biophys Acta.

[43] Yeh CH, Bellon M, Nicot C (2018). FBXW7: a critical tumor suppressor of human cancers. Mol Cancer.

[44] McCarty KS, Szabo E, Flowers JL, Cox EB, Leight GS, Miller L (1986). Use of a monoclonal anti-estrogen receptor antibody in the immunohistochemical evaluation of human tumors. Cancer Res.

[45] Dang TP, Gazdar AF, Virmani AK, Sepetavec T, Hande KR, Minna JD (2000). Chromosome 19 translocation, overexpression of Notch3, and human lung cancer. J Natl Cancer Inst.

[46] Monteith JA, Mellert H, Sammons MA, Kuswanto LA, Sykes SM, Resnick-Silverman L (2016). A rare DNA contact mutation in cancer confers p53 gain-of-function and tumor cell survival via TNFAIP8 induction. Mol Oncol.

[47] Young L, Sung J, Stacey G, Masters JR (2010). Detection of mycoplasma in cell cultures. Nat Protoc.

[48] Sun Y, Moretti L, Giacalone NJ, Schleicher S, Speirs CK, Carbone DP (2011). Inhibition of JAK2 signaling by TG101209 enhances radiotherapy in lung cancer models. J Thorac Oncol.

[49] Chang BD, Broude EV, Dokmanovic M, Zhu H, Ruth A, Xuan Y (1999). A senescence-like phenotype distinguishes tumor cells that undergo terminal proliferation arrest after exposure to anticancer agents. Cancer Res.

[50] Huck JJ, Zhang M, Mettetal J, Chakravarty A, Venkatakrishnan K, Zhou X (2014). Translational exposure-efficacy modeling to optimize the dose and schedule of taxanes combined with the investigational Aurora a kinase inhibitor MLN8237 (alisertib). Mol Cancer Ther.

[51] Huck JJ, Zhang M, McDonald A, Bowman D, Hoar KM, Stringer B (2010). MLN8054, an inhibitor of Aurora a kinase, induces senescence in human tumor cells both in vitro and in vivo. Mol Cancer Res.

[52] Kim HJ, Cho JH, Quan H, Kim JR (2011). Down-regulation of Aurora B kinase induces cellular senescence in human fibroblasts and endothelial cells through a p53-dependent pathway. FEBS Lett.

[53] Luo H, Yang A, Schulte BA, Wargovich MJ, Wang GY (2013). Resveratrol induces premature senescence in lung cancer cells via ROS-mediated DNA damage. PLoS One.

[54] Min YH, Kim W, Kim JE (2016). The Aurora kinase A inhibitor TC-A2317 disrupts mitotic progression and inhibits cancer cell proliferation. Oncotarget.

[55] Ferbeyre G, de Stanchina E, Lin AW, Querido E, McCurrach ME, Hannon GJ (2002). Oncogenic ras and p53 cooperate to induce cellular senescence. Mol Cell Biol.

[56] Shay JW, Pereira-Smith OM, Wright WE (1991). A role for both RB and p53 in the regulation of human cellular senescence. Exp Cell Res.

[57] Nadler Y, Camp RL, Schwartz C, Rimm DL, Kluger HM, Kluger Y (2008). Expression of Aurora a (but not Aurora B) is predictive of survival in breast cancer. Clin Cancer Res.

[58] Siggelkow W, Boehm D, Gebhard S, Battista M, Sicking I, Lebrecht L (2012). Expression of aurora kinase a is associated with metastasis-free survival in node-negative breast cancer patients. BMC Cancer.

[59] Sehdev V, Katsha A, Arras J, Peng D, Soutto M, Ecsedy J (2014). HDM2 regulation by AURKA promotes cell survival in gastric Cancer. Clin Cancer Res.

[60] Yang G, Chang B, Yang F, Guo X, Cai KQ, Xiao XS (2010). Aurora kinase a promotes ovarian tumorigenesis through dysregulation of the cell cycle and suppression of BRCA2. Clin Cancer Res.

[61] Goos ACM, Coupe VMH, Diosdado B, Delis-Van Diemen PM, Karga C, Belien JAM (2013). Aurora kinase a (AURKA) expression in colorectal cancer liver metastasis is associated with poor prognosis. Mol Diag.

[62] Ogawa E, Takenaka K, Katakura H, Adachi M, Otake Y, Toda Y (2008). Perimembrane Aurora-a expression is a significant prognostic factor in correlation with proliferative activity in non-small-cell lung cancer (NSCLC). Ann Surg Oncol.

[63] Lo Iacono M, Monica V, Saviozzi S, Ceppi P, Bracco E, Papotti M (2011). Aurora kinase a expression is associated with lung cancer histological-subtypes and with tumor de-differentiation. J Transl Med.

[64] Liu Y, Hawkins OE, Su Y, Vilgelm AE, Sobolik T, Thu YM (2013). Targeting aurora kinases limits tumour growth through DNA damage-mediated senescence and blockade of NF-κB impairs this drug-induced senescence. EMBO Mol Med.

[65] Islam S, Qi W, Morales C, Cooke L, Spier C, Weterings E (2017). Disruption of aneuploidy and senescence induced by Aurora inhibition promotes intrinsic apoptosis in double hit or double Expressor diffuse large B-cell lymphomas. Mol Cancer Ther.

[66] Ma ZL, Zhang BJ, Wang DT, Li X, Wei JL, Zhao BT (2015). Tanshinones suppress AURKA through up-regulation of miR-32 expression in non-small cell lung cancer. Oncotarget..

[67] Sun C, Chan F, Briassouli P, Linardopoulos S (2007). Aurora kinase inhibition downregulates Nf-kB and sensitises tumour cells to chemotherapeutic agents. Biochem and Biophys Comm.

[68] Kojima K, Konopleva M, Tsao T, Nakakuma H, Andreef M (2008). Concomitant inhibition of Mdm2-p53 interaction and Aurora kinases activates the p53-dependent postmitotic checkpoints and synergistically induces p53-mediated mitochondrial apoptosis alone with reduced endoreduplication in acute myelogenous leukemia. Blood..

[69] Liewer S, Huddleston A (2018). Alisertib: a review of pharmacokinetics, efficacy and toxicity in patients with hematologic malignancies and solid tumors. Expert Opin Investig Drugs.

[70] Damodaran AP, Vaufrey L, Gavard O, Prigent C (2017). Aurora a kinase is a priority pharmaceutical target for the treatment of cancers. Trends Pharmacol Sci.

[71] Tayyar Y, Jubair L, Fallaha S, McMillan NAJ (2017). Critical risk-benefit assessment of the novel anti-cancer aurora a kinase inhibitor alisertib (MLN8237): a comprehensive review of the clinical data. Crit Rev Oncol Hematol.

[72] D'Assoro AB, Haddad T, Galanis E (2016). Aurora-a kinase as a promising therapeutic target in Cancer. Front Oncol.

