# Supplementary material for: Inhibition of Aurora A enhances radiosensitivity in selected lung cancer cell lines
Source: Respir Res. 2019 Oct 23;20:230. doi: 10.1186/s12931-019-1194-8 (PMC6813099; doi:10.1186/s12931-019-1194-8)
Supplement: Supplementary file 1 — Additional file 1: Table S1. Association of AURKA expression with patient’s clinicopathologic characteristics in NSCLC. [file 12931_2019_1194_MOESM1_ESM.docx]

**Table S1 Association of AURKA expression with patient’s clinicopathologic characteristics in NSCLC**

|  |  | **AURKA expression n (%)** | | | |
| --- | --- | --- | --- | --- | --- |
| **Variable** | **Cases** | **Low (50)^a^** | **High (13)** | | **P value** |
| **Age (years)** |  |  | |  |  |
| ≥ 57 | 32 | 23 (46) | | 9 (69) | 0.3984 |
| < 57 | 31 | 27 (54) | | 4 (31) |  |
| **Gender** |  |  | |  |  |
| Female | 16 | 11 (22) | | 5 (38) | 0.2282 |
| Male | 47 | 39 (78) | | 8 (62) |  |
| **Pathology** |  |  | |  |  |
| SCC | 32 | 24 (48) | | 8 (62) | 0.3882 |
| ADC | 31 | 26 (52) | | 5 (38) |  |
| **Stage** |  |  | |  |  |
| IA-IB | 20 | 19 (38) | | 1 (8) | 0.0963 |
| IIA-IIB | 14 | 9 (18) | | 5 (38) |  |
| IIIA-IIIB | 26 | 19 (38) | | 7 (54) |  |
| IV | 3 | 3 (6) | | 0 |  |
| **LN metastasis** |  |  | |  |  |
| Positive | 29 | 20 (40) | | 9 (69) | 0.0617 |
| Negative | 34 | 30 (60) | | 4 (31) |  |

a: Four negative cases were included in the 50 “Low expression of AURKA” cases; SCC: Squamous cell carcinoma; ADC: adenocarcinoma, LN: Lymph node; data was analyzed by Prism Graphpad and double checked with SPSS 25 software.
